# Use of Graphene Oxide to Improve the Durability and Mechanical Properties of Mortar Immersed in Flowing River for Three Years

**DOI:** 10.3390/nano10122385

**Published:** 2020-11-29

**Authors:** Dong Cui, Hao Wei, Xiaobao Zuo, Keren Zheng, Qiannan Wang

**Affiliations:** 1 Department of Civil Engineering, School of Science, Nanjing University of Science & Technology, Nanjing 210094, China; 1520664820@njust.edu.cn (H.W.); xbzuo1968@163.com (X.Z.); 2 Department of Civil Engineering, Central South University, Changsha 410075, China; zhengkeren@csu.edu.cn; 3 School of Civil Engineering and Architecture, Zhejiang University of Science & Technology, Hangzhou 310023, China; wangqiannan@zust.edu.cn

**Keywords:** cementitious materials, graphene oxide, durability, microstructure, mechanical properties, permeability, porosity

## Abstract

Nanomaterials have received increased concentration in the field of civil engineering, as their incorporation can effectively modify the mechanical and transport properties of cementitious composites. In this study, to understand the effect of graphene oxide (GO) nanoparticles on the durability and mechanical properties of cementitious composites serving underwater, mortars incorporated with GO were taken for study. To match the real circumstance, all specimens were immersed directly in a flowing river for three years, and their transport properties, mechanical properties and microstructure before, and after, river experience were studied separately. The results showed that the incorporation of GO could reduce both early-age permeation coefficient and later-age chloride migration coefficient of mortar specimens. The average porosities of mortars could be reduced by the range of 3.37–11% with GO incorporation. Moreover, through a novel dual-scan method, GO incorporation was confirmed effective in enhancing both the leaching and cracking resistance. Furthermore, the compressive strengths, flexural strengths and splitting tensile strengths could be improved by the range of 4.37–9.82%, 7.78–22.33%, 8.14–28.73%, respectively with GO incorporation, and the tested mechanical strengths for GO-incorporated mortar after three-year river experience could be retained to a higher extent. Finally, based on durability and mechanical properties, the optimum mix proportion of GO was determined to be 0.06 wt.% in this study. The work presented here is of high reference value for the designing of marine infrastructure and can help promote the application of nanomaterials in civil engineering.

## 1. Introduction

In recent decades, the construction of bridges, dams, ports and tunnels, such as Hong Kong-Zhuhai-Macao bridge, Bohai bay cross-sea channel, Banpo port, etc., has been carried out massively in developing countries. All aforementioned projects are beneficial for the growth of the national economy and promotion of people’s living standard, and thus, are of high importance. However, during years of service underwater, the marine infrastructures have become vulnerable towards the actions of waves, undercurrents, tides and other kinds of waterflows, and these water actions can cause gradual deterioration on concrete durability, leading to shortened service life of the whole infrastructure [1,2,3]. Therefore, it is essential to understand the degradation pattern of durability for underwater concretes, as it can help offer reliable assessments on the infrastructure serving underwater. Moreover, with a systematic understanding of the degradation mechanism, it is possible to propose methods that can improve the durability performance of marine infrastructures.

Under long-term erosion by environmental water, the calcium ions inside the pore solution of concrete will gradually diffuse into the environmental water, leading to a decrease on the calcium-ion concentration inside pores [4]. Along the process, hydration products like calcium hydroxide (CH) and calcium silicate hydrate (C-S-H) will gradually dissolve, and the “leaching” process, deteriorates the microstructure of underwater concretes, causing increase on its open porosity [5]. Extensive former work has been performed to understand the underwater behavior of concrete, and now it is widely believed that leaching is the “culprit” behind the degradation of durability for concretes serving underwater [4,6,7]. Along the process, macroscopic properties, such as strength and impermeability, will gradually decrease as well [5,8,9]. Nonetheless, most former investigations concerning underwater performance of concrete were carried out under steady water in laboratory [8,9,10], and the effect of water action was overlooked. However, in real circumstances, marine infrastructures is vulnerable towards the actions of waves, undercurrents, tides and other water actions, and their effects on concrete durability are indeed non-ignorable. Momber et al. [11] used high-velocity waterflow to flush the surface of concrete, and the structures of concrete subjected to variant velocities of waterflow were studied. Based on Momber’s observation, the flushing force created by high-velocity waterflow could damage the concrete, causing propagation of the cracks near the concrete surface, especially within the areas between aggregates and hydrated cement. The pioneering work revealed the effect of water action on the degradation of durability for concrete, but high-velocity waterflow, adopted in that work, was not compatible to the water actions in real circumstances, and the effect of water action was, thereby, exaggerated. Hu et al. [12] embedded cylindrical mortar specimens in pipes, and used 1 M ammonium chloride solution of variant speeds to flush along the pipe. Both the microstructure and chemical composition of specimens before, and after, flushing were tested, based on which the effects of flowing solutions on concrete properties were studied. The results showed that, flowing solution with higher velocity was more detrimental to the durability of mortar specimen, as shear force, created by solutions with higher velocity, could introduce larger extent of damage to the tested specimen, and the damage could promote leaching further. Even though the work itself is useful, the simulated underwater environment still deviates from that in practical circumstance, as the water action in real circumstance is highly erratic. Therefore, to systematically uncover the mechanism of durability degradation for concrete serving underwater, carrying out in-site tests is indispensable.

Despite the deficiency, several protocols, enhancing the durability performance of marine concrete have already been accessible, based on former laboratory tests. For example, according to packing density theory, the durability and mechanical performance of concrete serving underwater can be promoted through blending of micro minerals (e.g., fly ash [6,13], silica fume [14]). Better yet, in recent years, with the development on nanotechnology, several nanomaterials, including nano-silica [15,16], carbon nanotube [17,18], nano-titanium oxide [19], sulfonated graphene [20,21], graphene [22,23,24,25], etc., have also been proven effective in controlling the durability and mechanical properties of concrete [26]. Among them, graphene oxide is receiving increasing attention due to following reasons: firstly, owning to close packing effect [27] and nucleation effect [28], the hydration degree of cementitious composites can be increased after GO incorporation, and that leads to a more compact structure with better mechanical and durability performances. Secondly, the GO nanoparticle is capable of tuning the morphology of cement hydration products [29], which can also contribute to the enhancement of mechanical and transport properties for concrete; and thirdly, graphene oxide itself has promising mechanical properties, with an elastic modulus as high as 300 GPa and an inherent strength as high as 112 GPa [30]. In addition, according to former investigations, the positive effects of GO could be identified when a low mix proportion of GO was applied [31], and with advanced nanotechnology, the cost of industrial scale of GO production has been largely reduced. Even so, limited former work focused on the effect of GO on the mechanical and durability performance of concretes serving underwater; also, almost all previous work was carried out in laboratory, the effect of water action on GO-incorporated cementitious composites was not considered, and the obtained degradation mechanism may, thus, be incompatible for the real underwater environments.

In this study, in order to clarify the degradation pattern of concrete durability serving underwater, instead of immersing test samples in steady solution, all samples were immersed directly in a flowing river for three years. To clarify the effect of GO-incorporation on the microstructure and transport properties of cementitious composites, four mix proportions of GO were adopted, and early-age water permeability test, chloride penetration test, as well as mercury intrusion porosimetry (MIP) test were performed respectively. Furthermore, to unveil the degradation mechanism of GO-incorporated concrete serving underwater, the spatial distribution of local porosity for all samples, after a three-year river experience, were studied through a novel dual-scan method, and the mechanical properties (compressive strength, tensile splitting strength and flexural strength) for all samples before, and after, three-year river experience were tested accordingly. The work can help explain the effect of GO incorporation on the mechanical and durability performance of cementitious composites serving underwater, and is therefore, of high importance in guiding the design of marine structure with adequate engineering performance. In addition, current work can help promote the application of nanomaterials in civil engineering.

## 2. Materials and Methods

### 2.1. Materials and Casting Strategy

**Figure 1:**
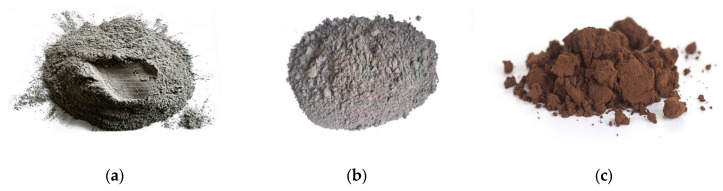
Typical photographs of raw materials. (**a**) Cement; (**b**) Fly ash; (**c**) Graphene oxide.

In this study, P⋅II 52.5 cement produced from Xiaoyetian cement factory (Dalian, China) and Type-F fly ash produced from Jianbi powerplant, Zhenjiang, China, were used (see Figure 1). The chemical compositions of both raw materials are listed in Table 1.

Quartz sand was used in this study with a diameter of less than 2 mm. Graphene oxide (GO) nanoparticles produced by powder research center in Southeast University, Nanjing, China, were used as addictive (see Figure 1), which properties are listed in Table 2.

The water content of sand significantly affects the durability and mechanical performances of concrete. Therefore, all sand was pre-conditioned through the following procedures: First, the sand was immersed in water for 12 h; second, sand was transferred to a dry area and conditioned at ambient temperature. The procedure could create sands with saturated core and dry surface, which were suitable for casting.

The water to binder ratio was 0.3, the sand to binder ratio was 0.5, and 30 wt.% of the cement was replaced by fly ash for all mix proportions (see Table 3 for the detailed mix proportions). The blending of fly ash reduces the amount of cement required for casting, and the cost for construction can therefore be reduced; besides, fly ash is the byproduct of coal industry, so it can serve as eco-friendly construction material. GO suspension was prepared through 3 h of ultrasonic dispersion. Next, the solution was mixed with cement, fly ash and sand. Since the water to binder ratio was 0.3 in this study, the fresh pastes were of good workability. For early-age water-penetration tests, the specimens were cast in cylindrical molds with a diameter of 100 mm and a length of 200 mm. The central part of the mold was occupied by a PVC cylinder with a diameter of 73 mm and a length of 200 mm. Therefore, hollow cylindrical specimens were cast, and the specimens were de-molded after 24 h, prepared for water penetration tests.

For compressive and splitting tensile strength tests, all samples were cast in molds with size of 100 mm × 100 mm × 100 mm. For flexural strength tests and CT scans, samples were cast in molds with size of 100 mm × 100 mm × 400 mm. After casting, all molds were wrapped instantly by plastic film and were left at ambient condition for 24 h. Next, all specimens were de-molded, and were further cured under standard curing condition (20 ± 1 °C and RH > 95%) for 28 more days.

**Figure 2:**
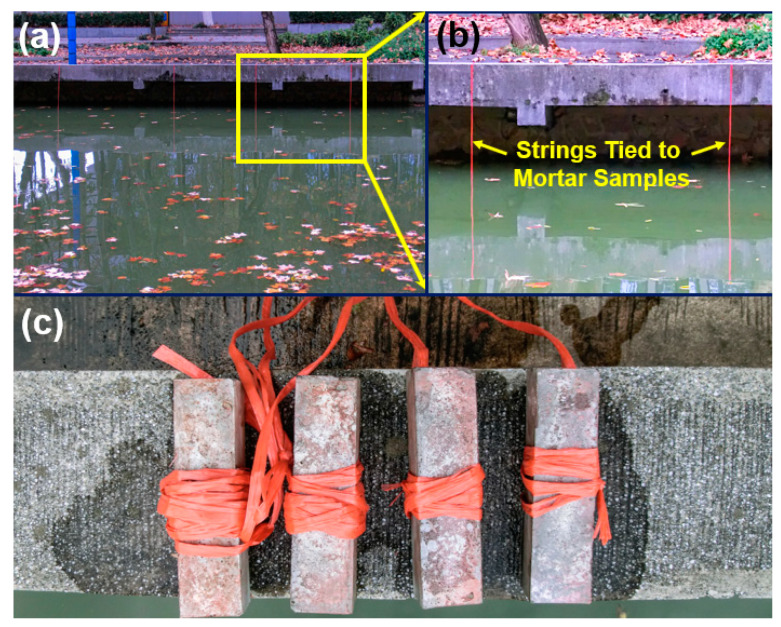
Illustration of the sites where mortars were immersed. (**a**) Test locations along Jiulonghu riverside; (**b**) Magnified photographs of the strings tied to mortar samples; (**c**) Typical samples after river immersing.

After 28 d of standard curing, samples were hanged by strings and were immersed 1.5 m below the water surface of Jiulonghu river surrounding Jiangning Campus, Southeast University for three years (see Figure 2). Even though the water level of the river fluctuated seasonally, all mortars were maintained underwater throughout the test period. The samples before, and after, the three-year river experience were taken respectively for microstructure and mechanical tests.

### 2.2. Experimental Methods

#### 2.2.1. Water-Permeation Test

**Figure 3:**
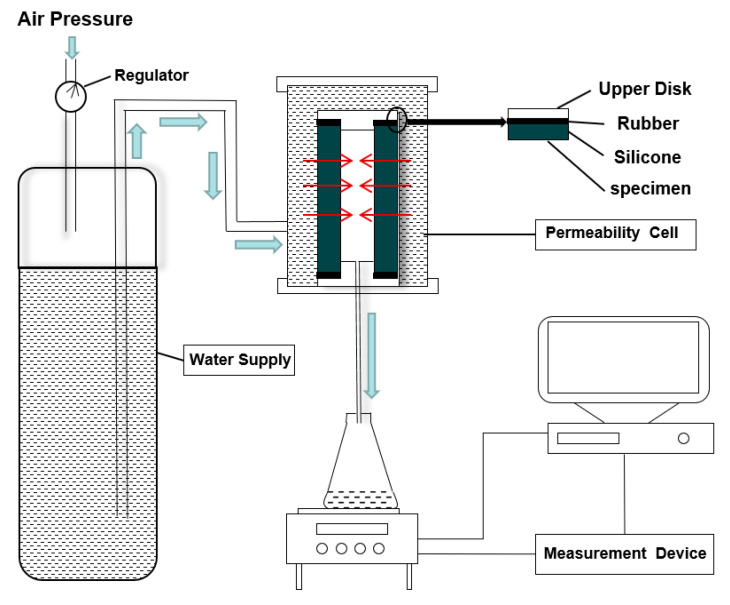
Schematic diagram for the apparatus testing water permeation coefficient (Reproduced with permission of [33]. Springer-Verlag, 2018).

For testing the early-age water-permeating property, it was conducted based on the homemade apparatus developed by Bhargava and Banthia [32] (see Figure 3 for the schematic of the testing apparatus). A constant inflow water with a pressure of 0.35 MPa was adopted for all tests, which was in accordance with that in Reference [33]. Since the water pressure occupied merely 3% of the compressive strength for the tested specimens at 48 h, no deleterious effect on the properties of the tested specimen was supposed to occur.

The mass of the water permeated through the permeability cell was collected and weighed as a function of time. The tests were carried out 28 h after casting. By applying Darcy’s law, the coefficient of water permeability can be deduced, as shown in Equation (1) [34],

(1) $$K_{w}=\frac{Q\times \ln\left(\frac{d_{2}}{d_{1}}\right)}{2\pi h\times \Delta H}$$

where, $K_{w}$ represents the permeability coefficient of water (m/s); Q represents the rate of water flow (m^3^/s); $d_{1}$ and $d_{2}$ represent the internal and external diameter of the tested specimen (m), respectively; h represents the height of the specimen (m), and ΔH represents the difference of hydraulic heads between the internal and external sides of the specimen (m).

Equation (1) works when an equilibrium condition has been reached inside the testing system. Consistent with former work [33,35], one hour was assumed for the establishment of equilibrium, and all experimental data were recorded after that. To be representative, for each mix proportion, ten specimens were tested, and the averaged results were recorded.

#### 2.2.2. Chloride Penetration Test

Due to rigid hydration, the water penetration test can only be applied to young specimens. Therefore, chloride penetration test was applied to examine the transport properties at later curing ages. The test was performed after 28 d of curing following Chinese Standard “GB/T 50082-2009” [36], where cylindrical specimens with a diameter of 100 mm and a height of 50 mm were adopted. To guarantee the reproducibility of the testing results, for each mix proportion, five specimens were taken for tests, and the averaged result was taken as the chloride penetration coefficient for each mix proportion.

#### 2.2.3. Mercury Intrusion Porosimetry (MIP) Tests

Mercury Intrusion Porosimetry (MIP, Micromeritics, Shanghai, China) test was used to investigate the open porosity and pore size distribution of the tested specimen after 28 d of curing. The machine was one Autopore 9500 manufactured in China. During the tests, the contact angle was assumed as 130°, and testing pressure ranged from 242 MPa down to 0.003 MPa, which covered pore radii ranging from 3 nm to 180 μm. For each mix proportion, three specimens were taken for tests, and the averaged results were recorded.

#### 2.2.4. Computed Tomography (CT) Tests

Computed tomography (CT, YXLON, Hamburg, Germany) was adopted in the present study to investigate the microstructure for mortar immersed in river. Serving as typical non-destructive testing (NDT) method, CT has not received adequate attention in the field of concrete research mainly due to its limited resolution [37]. To mitigate the deficiency, several researchers, including the authors here, attempted to propose novel methods which can enhance the precision level of CT [37,38]. One author recently developed a method, denoted as “dual-scan” method, and adopted an altered local attenuation coefficient during the dual CT scan, instead of local attenuation coefficient from a single CT scan to measure the local porosity of a scanned object [39]. Since water was used as the intrusion agent in dual CT scan, the covered range of pores could be even broader than mercury intrusion porosimetry (MIP) [40]. For that reason, dual-scan method was adopted in this study, and its basic testing procedures are as follows:

First, partly leached specimens were sealed with epoxy resin to protect the vulnerable surface. Then, the specimens were cut into slices with a thickness of 5 mm, which aimed to accelerate the saturation and drying processes. Next, the sliced sample was conditioned in vacuum oven under 45 °C for two weeks, by which time the mass of the sample no longer changed, and the dried sample was then taken immediately for the first-time CT scan. Finally, the dried sample after scanning was immersed in boiled and cooled water for two weeks, and with the assistance of a 1.5 CFM working force pump, the air in pores of the tested sample was substituted completely by water. The saturated sample was taken immediately for the second-time CT scan.

Combining registration technique, the CT data from dual scans can be geometrically matched. Besides, the scanning conditions for all scans were hold still, so the local porosity within each voxel could be deduced as [38],

(2) $$W=\frac{G_{\mathrm{sat}}-G_{\mathrm{dry}}}{G_{\mathrm{water}}-G_{\mathrm{air}}}\times 100\%$$

where $G_{\mathrm{sat}}$ represents the gray-scale value (GSV) of a voxel from saturated specimen; $G_{\mathrm{dry}}$ represents the GSV of the same voxel from dried specimen; $G_{\mathrm{water}}$ represents the GSV of water; and $G_{\mathrm{air}}$ represents the GSV of air, which was null in this study.

YXLON Company in Germany produced the CT used in this study. A peak energy of 195 kV and a working current 0.3 mA were adopted during our experiments. The acquisition time of 500 ms was set for each projection, and six routines were averaged to increase the signal to noise ratio. Finally, the effective resolution for all scans was set as 60 μm.

#### 2.2.5. Mechanical Tests

Compressive tests and splitting tensile tests were carried out via TYA-2000 electro-hydraulic pressure testing machine (Xinluda, Wuxi, China), and flexural tests, with loading span of 180 mm and supporting span of 360 mm were carried out via CMT5105 testing machine (Xinsansi, Shenzhen, China). The loading rate for all mechanical tests was set as 0.08 MPa/s. To guarantee the reliability of the testing results, eight samples were prepared for each test, and the average results were recorded.

## 3. Results and Discussion

### 3.1. Early-Age Water Permeation Coefficient

**Figure 4:**
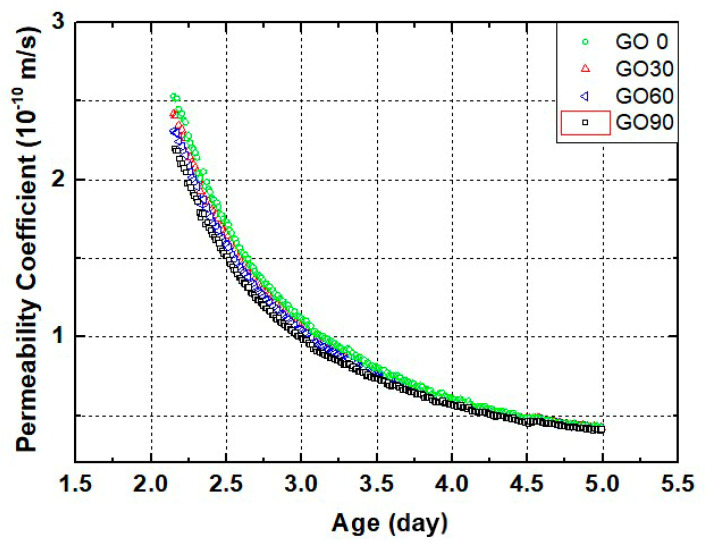
Permeability plots of GO-incorporated mortars by the curing age of 2–5 day.

Figure 4 shows the curves of early-age water permeability coefficient for mortars containing different amounts of GO. The test was restricted to the first few days of curing because the permeability coefficient for young specimens reduced sharply during the period, and the permeability coefficient could no longer be precisely measured after that. Also, it should be noted here, the microstructure of mortar specimen at early ages was quite fragile, and several movements such as casting, demolding and transferring could all cause cracking inside the sample. Therefore, the permeability coefficient curve obtained from one sample showed high randomness. To be representative, for each curve presented on Figure 4, they are the average results measured from ten independent samples.

As shown in Figure 4, the average permeability coefficients of all four groups were approximately $2.3\times 10^{-10}$ m/s by the curing age of 2 d, while reduced to approximately $4\times 10^{-11}$ m/s by the curing age of 5 d. The obtained results agreed with the results obtained by Wang et al. [35]. The permeability coefficient tended to be high at 2 d, while reduced shapely during latter test period for all four groups. The results highlighted the ongoing rigid hydration in all young specimens, which led to significant truncation in the permeability paths and increased tortuosity in cracks [41]. Hydration was capable of densifying the microstructure of the tested specimen, and that could also cause reduction on water permeability [42]. Furthermore, internal healing of initial cracks or defects could also contribute to the reduced permeability coefficient [43].

Even though the early-age permeation coefficients appeared similar for all groups, the effect of GO on early-age permeation coefficient could be identified. As shown in Figure 4, at the curing age of 2 day, the permeability coefficient reduced slightly with increased GO content. The sequence was GO0 > GO30 > GO60 > GO90. The results confirmed the positive effect of GO incorporation in confining the early-age transport behaviors of cementitious composites, and the most plausible reason behind this reduction was that, in GO-incorporated groups. The GO nanoparticles could block the permeation paths and increased the tortuosity of the cracks, which led to a reduction in the permeation coefficient of the whole specimen [23,31].

Overall, the difference on permeation coefficient was limited at early curing age, and the difference on permeability coefficient between four groups was no longer observed by the curing age of 5 day, mainly due to limited precision level of current testing technique. Therefore, to quantify the transport properties for GO-incorporated mortars at later curing age, chloride penetration tests were performed.

### 3.2. Chloride Penetration Resistance

**Figure 5:**
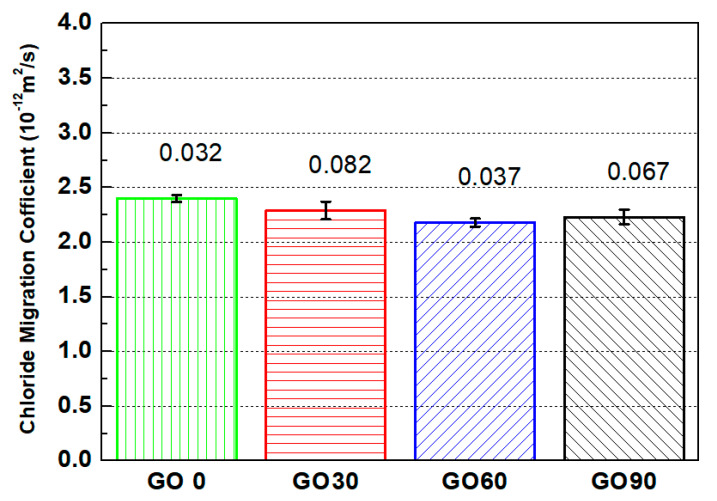
Chloride migration coefficients for mortar specimens containing different amount of GO.

Figure 5 shows the chloride penetration resistance for samples containing different amounts of GO. As shown in the figure, the chloride penetration coefficients for GO0, GO30, GO60 and GO90 were 2.41 $\times \;10^{-12}$ $m^{2}\cdot s^{-1}$, 2.29 $\times \;10^{-12}$ $m^{2}\cdot s^{-1}$, 2.18 $\times \;10^{-12}$ $m^{2}\cdot s^{-1}$ and 2.23 $\times \;10^{-12}$ $m^{2}\cdot s^{-1}$, respectively. The migration coefficient of chloride was approximately two order of magnitude lower compared with the permeation coefficient of water by the curing age of 2 d, which was within our expectation, as the microstructure of the specimen became denser after longer term of curing. Compared with GO0 (the reference group), the chloride penetration coefficients of GO30, GO60 and GO90 were reduced by 4.98%, 9.54% and 7.47%, respectively. The results verified again that GO incorporation was beneficial in reducing the transport properties of cementitious composites, and two reasons could be referred here as the explanation. First, similar to early-age behavior, GO incorporation was capable of blocking the permeation paths and increasing the tortuosity of cracks, so the migration coefficient of chloride would spontaneously be lower. Second, GO nanoparticles were capable of promoting the hydration degree of cementitious composites, and that also leaded to a more densified microstructure [29,42], so the permeation coefficient of the specimen containing GO would be reduced.

Based on the chloride penetration resistance, the optimum GO content was determined as 0.06 wt.%, as the lowest chloride penetration coefficient was identified in GO60. The result agreed with that reported earlier [31], and, in line with former work, improper dispersion (even agglomeration) of GO nanoparticles at high mix proportion (0.09 wt.% in this study), which led to a more porous microstructure, and further re-increased chloride penetration coefficient from GO60 to GO90, was referred here as the explanation.

### 3.3. Average Porosity and Pore Size Distribution

**Figure 6:**
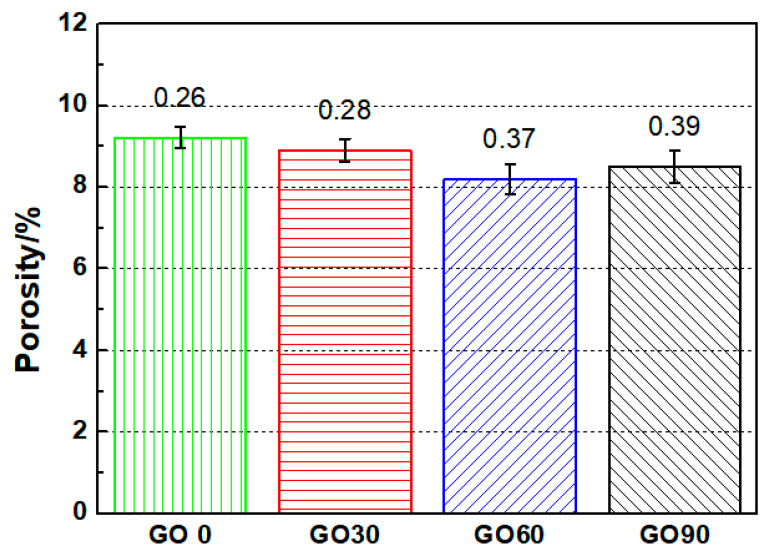
Average porosities for mortar specimens containing different amount of GO.

**Figure 7:**
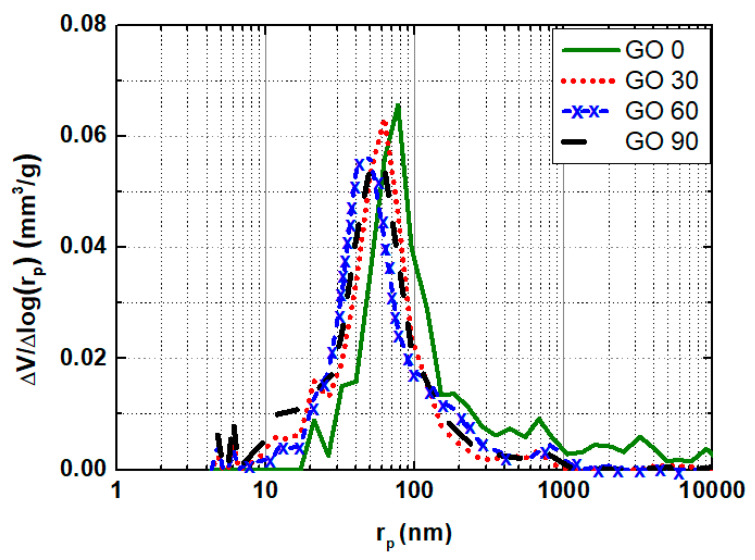
Pore size distribution curve for mortar specimens containing different amount of GO.

To uncover the microstructure evolution for specimens containing different amounts of GO, mercury intrusion porosimetry (MIP) tests was performed on specimens at the curing age of 28 d. The obtained porosity as well as pore size distribution curves are presented in Figure 6 and Figure 7, respectively. Note that the porosity by MIP was measured mainly from capillary pores (open porosity), which controlled the chloride migration properties of mortars in this study.

As shown in Figure 6, the average porosity of GO0, GO30, GO60 and GO90 were 9.2%, 8.9%, 8.2% and 8.5%, respectively. With the addition of GO nanoparticles, the average porosity of the tested specimen was reduced, which verified that GO incorporation was beneficial in refining the microstructure of cementitious composites. The porosity results showed consistency with the results obtained through chloride penetration test, and similar with the explanation on transport behaviors, promotion on the hydration degree of the test specimen after GO incorporation was taken as the main cause for the reduced porosity. The average porosity for GO30, GO60 and GO90 were 3.37%, 11% and 7.71% lower as compared to GO0, based on which the optimum mix concentration of GO was selected to be 0.06 wt.% regarding average porosity.

To further illustrate the full image on pore structure for mortars containing different amounts of GO, the pore-size distribution maps for all four groups were plotted as well, as shown in Figure 7. In general, the pore-size distribution curves for all testing groups appeared similar, with a singular peak representing the most probable pore radius. Despite the similarity, the location of the peak varied among four groups: for GO0, GO30, GO60 and GO90, the most probable pore radii were approximately 80 nm, 60 nm, 40 nm, and 50 nm, respectively. The most probable pore radius did not keep decreasing with increased mix proportion of GO, as from GO60 to GO90, the most probable pore radius regained an increase. The obtained trend here was in accordance with the trend reported elsewhere [44], and that validated the existence of optimum mix concentration of GO (0.06 wt.%) regarding microstructure.

As a conclusion, GO incorporation was proven effective in refining the microstructure of cementitious materials. Also, the incorporation of GO was beneficial in reducing the water permeation and chloride migration behaviors of cement-based materials. To further evaluate the durability performance of GO-incorporated specimens serving underwater, the tested specimens were immersed in a flowing river for three years, and the microstructure of the specimen after three-year river experience was investigated. In addition, the mechanical strengths of the tested specimen before and after three-year river experience were compared, based on which the effect of GO incorporation on the durability and mechanical strength was studied accordingly.

### 3.4. Spatial Distribution of Local Porosity

#### 3.4.1. Mortars before, and after, River Immersing

**Figure 8:**
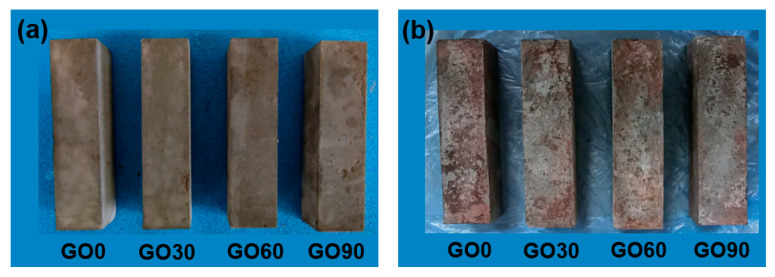
Mortars before and after river experience. (**a**) Specimens cured for 28 days; (**b**) Specimens immersing in river for 3 years.

Figure 8 shows typical mortars before and after immersing in river for three years. As illustrated in the figures, the sample surfaces were intact after 28 days of curing, but showed significant signs of erosion after immersing in river for three years. Nevertheless, no spalling was observed for all tested specimens after river experience, which suggested that the degradation degree of mortars was lower compared to accelerated leaching tests carried out in a laboratory [37]. Two reasons were given here to explain the lower degradation degree in this study: First, all samples were immersed permanently underwater, and wet-dry cycling, which caused serious cracking or even spalling of mortar, was thus avoided in this study; second, unlike leaching test performed in laboratory, water, instead of accelerated leaching solution, was used in this study, and the leaching process was thereby, milder.

Mass-loss tests were performed then. For all mortars, compared with the initial mass after 28 days of standard curing, the mass loss was less than 5% after immersing in river for three years. Aside from the lower degradation degree in this study, another reason should be noted: the mortars were exposed to a river environment for three years, so the specimens had gone through continuous absorption of water, the mass of absorbed water could counterbalance part of the mass loss due to leaching.

In general, Figure 8 could only reflect the surface status of the tested samples. To unveil the inside microstructure of all mortars, CT scan was required.

#### 3.4.2. Raw CT Data

**Figure 9:**
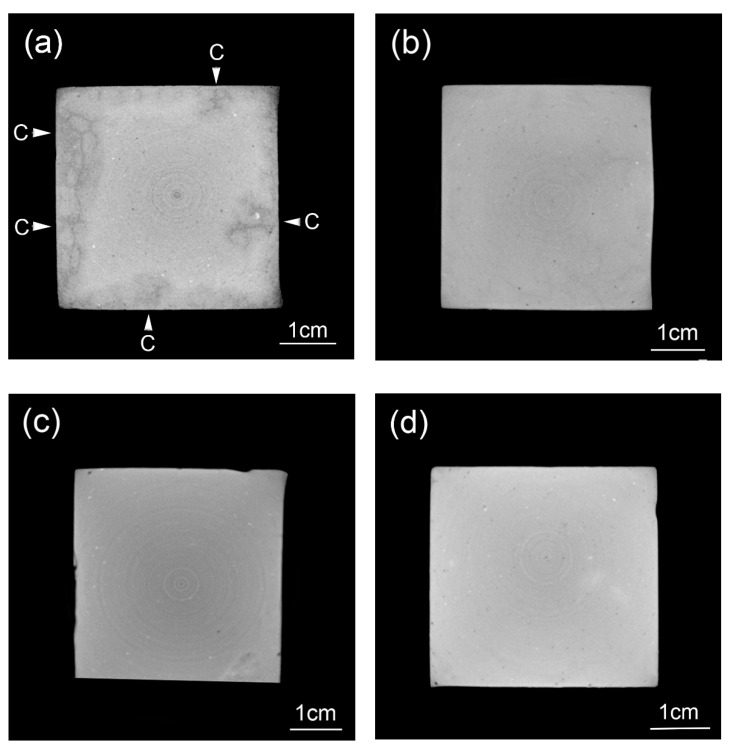
Typical renderings of cross-sectional areas for mortar specimens containing different amount of GO: (**a**) GO0; (**b**) GO30; (**c**) GO60 and (**d**) GO90. C: surface cracking.

Figure 9 shows typical cross-sectional images for specimens, containing different amounts of GO. The scanning was performed on the mortars after immersing in flowing river for three years. As shown in Figure 9a, the boundary of the samples was slightly darker as compared to the core area, which verified the existence of leaching: The leaching process involved the dissolution of hydration product, and that leaded to a reduction on the local attenuation coefficient within the partly leached area [45]. Since CT images were mapped from spatial distribution of local attenuation coefficient, the reduction on local attenuation coefficient resulted in darker local area. All leaching fronts presented in Figure 9 were blur, which was different from that reported elsewhere [45]. The main cause for the unclear boundary was that, the test in this study was performed in flowing river, not in accelerated tests, and the leaching process here was therefore, milder. Since the local leaching extent was relatively lower as compared to that based on accelerated leaching tests, the reduction on local attenuation coefficient was relatively smaller.

Comparing Figure 9a–d, the incorporation of GO was proven beneficial, as nearly no leaching fronts were observed in GO60 and GO90, highlighting better leaching resistance for groups containing GO.

In general, raw CT data could only offer qualitative results on the evolved microstructure, and the displayed results were vague. Therefore, in order to quantitatively illustrate the degradation behavior of GO-incorporated specimen serving underwater, advanced scanning technique (e.g., dual CT scan method) was required.

#### 3.4.3. Spatial Distribution of Local Porosity Based on Dual CT Scan

**Figure 10:**
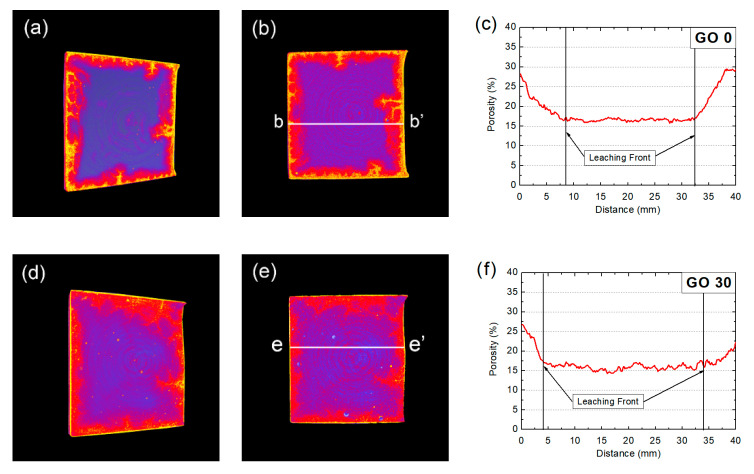

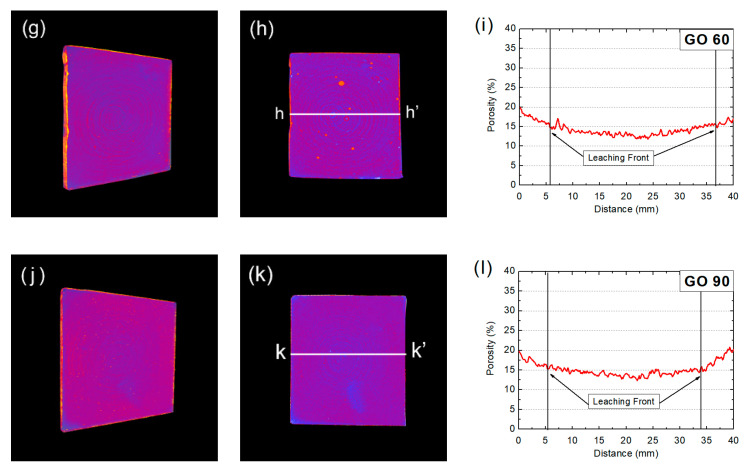
3D, 2D and 1D renderings on local porosity for mortars after 3-year river experience: (**a**,**d**,**g**,**j**): 3D renderings on local porosity for GO0, 30, 60 and 90, respectively; (**b**,**e**,**h**,**k**): 2D renderings on local porosity for GO0, 30, 60 and 90, respectively; (**c**,**f**,**i**,**l**): 1D profiles on local porosity for GO0, 30, 60 and 90, respectively.

Figure 10 shows three-dimensional (3D), two-dimensional (2D) and one-dimensional (1D) distributions of local porosity for specimens after the three-year river experience. The result was obtained through a dual scan of the tested mortar in the saturated state and in the dried state, and the local porosity was obtained through the altered attenuation coefficient during dual scans. The 3D and 2D renderings were displayed in the temperature map format, which meant a higher local porosity, as well as a brighter CT rendering area appeared. For linear distribution of local porosity (1D profile), it was plotted through linear scan of the related 2D porosity rendering. Since leaching leaded to the dissolution of hydration products, which further caused increased local porosity, the spatial renderings of local porosity could be used to identify the leached areas.

As shown in Figure 10, the leached area could be more clearly identified, compared to raw CT data (see Figure 9), which was because of the fact that the difference based on local porosity between leached area and non-leached area was much larger as compared to that based on gray scale value (GSV). Also, judging from Figure 10, the leaching resistance of GO0 and GO30 was lower as compared to GO60 and GO90, because significant leaching areas were observed in Figure 10a,d, while nearly no leaching front was present in Figure 10g,j. Comparing Figure 10a,d, the leached area for GO30 appears smaller than that for GO0, and the boundary area of GO30 was darker (suggesting lower local porosity), both results validated that the leaching resistance of GO30 was better than that of GO0. The results showed intuitive and compelling evidence that incorporation of GO was beneficial for controlling the leaching rates of cementitious composites serving under water. In addition, restricted to the range of mix proportion in this study, higher concentration of GO seemed to be more effective.

In Figure 10, all rendered 3D and 2D leaching fronts were of irregular shape. A case in point was GO0, where irregular-shaped leaching front, with bright cusps pervading was present. The results showed clear difference in the leaching fronts, rendered earlier by Wan et al. [45], but to some extent, similar with the carbonated fronts rendered earlier by the same authors [40]. In accelerated carbonation tests, specimens were required to be pre-conditioned in oven, aimed at removing excessive water, and the process would inevitably leave thermal damage (cracking) near the sample surface. During the latter carbonation process, the CO_2_ was preferable to diffuse through the surface cracks, and that leaded to formation of irregular-shaped carbonation front. In terms of leaching, since thermal pre-treatment was no longer necessary, specimens were expected to acquire a more intact initial structure after preconditioning, and the chance to form an irregular-shaped leaching front would be much lower. Nonetheless, in the present study, irregular-shaped leaching front was observed, which implied that, despite the absence of thermal pre-treatment, other factors, such as shear force created by water flowing, temperature fluctuation due to seasoning, might also introduce damage to the sample surface. During three-year river experience, preferable leaching through the crack paths could lead to generation of leaching cusps, which resulted in the formation of irregular-shaped leaching fronts. The results here highlighted the difference on degradation pattern for specimens tested in laboratory and specimens tested in-site, and that further validated the importance of carrying out degradation tests on durability under real circumstance. It could further be deduced here, with GO incorporation, due to higher hydration degree and more densified microstructure, the mortar specimen tended to be more resistant toward cracking, and that should also contribute to the slower degradation of durability for cementitious composites serving under water.

1D profiles of local porosity were also plotted and shown in Figure 10. For all groups, the local porosity of non-leached area was approximately 15%, while the local porosity for the leached area increased to the range of 20–30%. The porosity for the leached area did not surpass 30%, which appeared lower than that reported in Reference [45]. The result demonstrated that, the leaching occurred in this study was incomplete, and local leaching extent here was lower as compared to that based on accelerated leaching tests. Meanwhile, the porosity for non-leached areas, obtained through dual-scan method, was larger than that obtained through MIP, which was due to different ranges of pores taken for measurement between two techniques. In MIP, since mercury was used as the intrusion agent, due to non-filtration nature of mercury, mainly capillary pores were captured; while for the dual-scan method, water was used as the intrusion agent, broader range of pores (including part of gel pores) could also be measured, so the measured local porosity was larger [38,45,46]. Strictly speaking, even though the porosity obtained by dual CT scan was larger than that by MIP, it did not cover all pores inside the tested mortars [40]. However, considering that impermeable pores only occupied a much smaller proportion as compared to permeable pores, the porosity results here could still be used to evaluate the mechanical and transport properties of mortars in the present study.

The porosity profiles presented large leaching depths for GO0 and GO30, while relatively smaller leaching depths for GO60 and GO90, which proved once more that the leaching resistance of GO-incorporated groups was better than the groups containing no or less amount of GO. In particular, even though the leaching reaction could hardly be observed for GO60 and GO90 on 3D and 2D renderings, the existence of leaching could be confirmed in 1D profiles (see Figure 10i,l), as the local porosity near sample surface was higher as compared to core areas for GO60 and GO90. Also, judging from the smaller leaching fronts identified on GO60 as compared to GO90, an optimum concentration for GO incorporation could still be determined as 0.06 wt.% regarding leaching resistance.

### 3.5. Mechanical Properties

In this study, mechanical properties like compressive strength, flexural strength and splitting tensile strength for specimens before and after three-year river experience were taken for study. Based on the data, the effect of GO incorporation on the mechanical properties could be assessed; also, through investigating the remaining mechanical strengths for mortars after river experience, the effect of GO incorporation on durability properties could be evaluated as well.

#### 3.5.1. Compressive Strength

**Figure 11:**
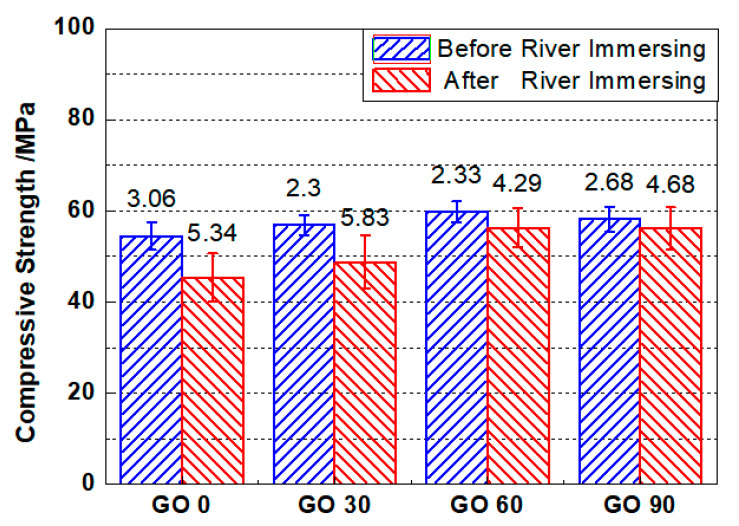
Compressive strengths for mortars containing different amount of GO before and after 3-year river experience. Error bar stands for the standard deviation.

Figure 11 shows the compressive strengths of GO0, GO30, GO60 and GO90 before and after three-year river experience. As shown in the figure, after 28 d of standard curing, the compressive strength of specimens incorporating GO was invariably higher, compared to specimens containing no GO. To be detailed, the compressive strengths of GO0, GO30, GO60 and GO90 were 54.46 MPa, 56.84 MPa, 59.81 MPa, and 58.13 MPa, respectively. The results revealed that the incorporation of GO was effective in enhancing the compressive strength of mortar. The enhanced compressive strength was believed to be caused by a densified microstructure. On one hand, GO nanoparticles could fill the pores of cementitious materials, leading to densified microstructure. On the other hand, GO nanoparticles could offer nucleation sites for cement hydration, and a refined microstructure could thus be formed due to higher hydration degree [47] and modified hydration products [48]. Compared with the compressive strength of GO0, the increased extents of compressive strengths for GO30 and GO60 were 4.37%, and 9.82%, respectively, but the increased extent fell back to 6.73% for the GO90. The result suggested that excessive incorporation of GO was improper for the strength development, and that showed consistency with the chloride penetration resistance tests and MIP results.

In Figure 11, the compressive strengths for mortar specimens after three-year river immersing were presented as well. Both GO0 and GO 30 suffered from significant loss on compressive strengths after river experience, but for GO60 and GO90, the compressive strengths were nearly retained. To be detailed, the compressive strength of GO0, GO30, GO60 and GO90 after river experience were 45.36 MPa, 48.76 MPa, 56.29 MPa and 56.07 MPa, respectively. Compared with the strength before river experience, the compressive strength was reduced by 16.7%, and 14.21%, respectively for GO0 and GO30, but almost unchanged for GO60 and GO90 (reduced by 5.89%, and 3.54%, respectively for GO60 and GO90). The results suggested significant extents of leaching in GO0 and GO30, and that could be supported by the porosity results revealed through dual CT scan (see Figure 10). For GO60 and GO90, even though the compressive strengths were almost retained after river experience, it did not necessarily mean that the degradation degrees were extremely low in both groups; during three years’ experience in river, further curing may compensate part of the strength loss, and the deterioration extent was therefore, underestimated. Finally, the error bars for groups after three-year river experience were universally larger as compared to that before, which implied that, for each mix proportion, the deterioration extents between tested specimen were inconsistent. One possible reason was given here for the greater errors after river experience, aside from leaching, other factors like water action or weathering, may also contribute to the reduced compressive strength. Since these environmental factors were highly erratic from place to place, the degradation degree varied even within identical mix proportion.

In this study, the obtained eight compressive strengths of each mix proportion could be fitted into normal distribution, and Student’s *t* test was, thus, applicable in examining the significances of difference between mortars incorporated with different amounts of GO.

Table 4 shows the significances of difference between the reference group (GO0) and the groups containing different amounts of GO. As shown in the table, the significance was close to 0.05 comparing GO0 and GO30, which suggested that the change regarding compressive strength was not obvious when low amount of GO (0.03 wt.%) was incorporated. The result was reasonable considering that with low amount of GO, the improvement on flexural strength was not significant. For the other comparisons, the significances were smaller than 0.05, which revealed that higher mix proportion of GO (larger than 0.03 wt.%) would significantly improve the compressive strengths for mortars both before and after river immersing. Furthermore, the minimum significance of difference was attained examining the difference between GO0 and GO60, based on which the optimum concentration for GO regarding compressive strength was determined to be 0.06 wt.%.

As a conclusion, adding GO is beneficial in enhancing the compressive strength of standard-cured mortar. A suitable amount of GO incorporation can help retain the compressive strengths of mortar after years of river experience. Finally, based on compressive strength, the optimum concentration for GO addition was determined to be 0.06 wt.% in our study.

#### 3.5.2. Flexural Strength

**Figure 12:**
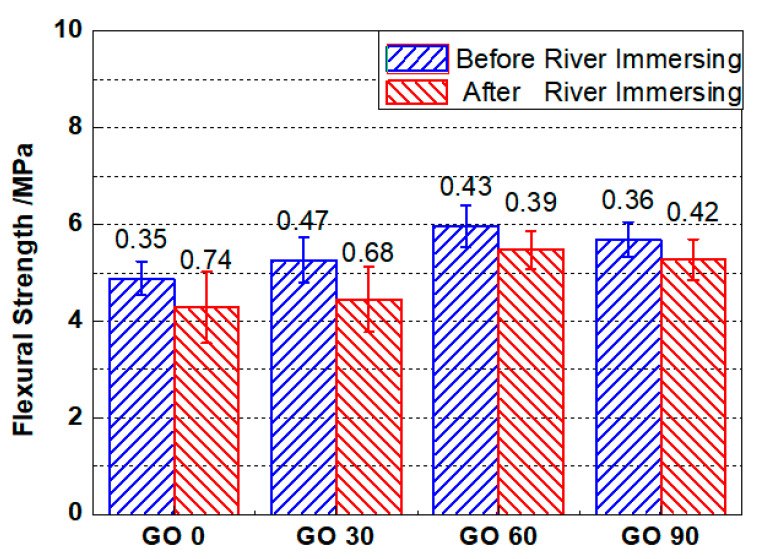
Flexural strengths for mortars containing different amount of GO before and after 3-year river experience. Error bar stands for the standard deviation.

Figure 12 shows the flexural strengths of testing specimens based on four-point flexural tests. The flexural strength of were 4.88 MPa, 5.26 MPa, 5.97 MPa, and 5.69 MPa respectively for GO0, GO30, GO60 and GO90. The flexural strength for each group was approximately 1/10 of its compressive strength, which was similar with the earlier reports [31]. The incorporation of GO was positive in enhancing the flexural strength of the cementitious composites, as the flexural strengths of GO30, GO60 and GO90 were 7.78%, 22.33%, and 16.6% respectively higher, compared with that of GO0. The revealed trend on flexural strength matched that based on compressive strength tests, and the optimum concentration of GO was referred as 0.06 wt.%, under which concentration the flexural strength attained its climax. Similar with the explanation earlier [47], addition of nanomaterials could promote cement hydration as well as densify the microstructure, both of which were beneficial for the increase of flexural strength for cementitious composites. As for GO90, the reduced flexural strength as compared to GO60 was most likely caused by improper dispersion (agglomeration) of GO nanoparticles.

After three-year river experience, all four groups suffered from loss on the flexural strength. The flexural strengths after river experience were 4.28 MPa, 4.45 MPa, 5.47 MPa, and 5.27 MPa respectively for GO0, GO30, GO60 and GO90. Unlike compressive strength results, the flexural strengths for GO60 and GO90 no longer retained after river experience. The results supported the earlier deduction: even though the compressive strengths after river experience were retained for GO60 and GO90, the deterioration extent of the samples still increased after river immersing. Nevertheless, the reduced extent for GO0 and GO30 were 12.3%, and 15.4%, respectively, compared to that before river experience, while the reduced extent for GO60 and GO90 were 8.38%, and 7.38%, respectively, compared to that previously. The smaller reductions on flexural strengths for GO60 and GO90 still revealed lower deterioration degree as compared to GO0 and GO30, which attested that GO incorporation was effective in slowing the degradation process of cementitious composites serving underwater, and the service life of marine infrastructure could be prolonged. Finally, based on the original and remaining flexural strengths, the optimum mix proportion of GO was selected to be 0.06 wt.%.

Similar normal distribution and Student’s *t* test were performed accordingly, and the significances of difference regarding flexural strength were given in Table 5. The revealed trend was similar with that in Table 4, where significant improvement on flexural strength was observed when the incorporated GO concentration was higher than 0.03 wt.%. The optimum mix proportion was determined to be 0.06 wt.%, as the smallest significance of difference attained when comparing GO0 and GO60.

#### 3.5.3. Splitting Tensile Strength

**Figure 13:**
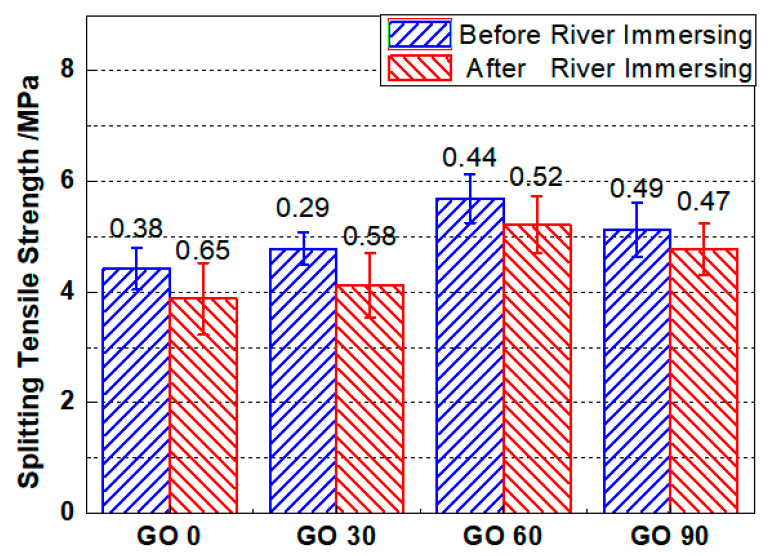
Splitting tensile strengths for mortars containing different amount of GO before and after 3-year river experience. Error bar stands for the standard deviation.

Figure 13 shows the splitting tensile strength of mortar specimens before and after three-year river experience. The splitting tensile strengths for GO0, GO30, GO60 and GO90 were 4.42 MPa, 4.78 MPa, 5.69 MPa, and 5.13 MPa, respectively. Compared with GO0, the improved tensile splitting strengths after GO incorporation ranged between 8.14% and 28.73%. The obtained splitting tensile strengths were slightly lower as compared to the flexural strength of the related group. Similar with the evolving pattern for compressive strength and flexural strength, when the incorporation content of GO was less than 0.06 wt.%, the splitting tensile strengths for the specimens containing GO kept increasing as the incorporated GO content increased. However, when the mix proportion reaches 0.09 wt.%, the splitting tensile strength of the tested specimen reduced slightly, but still higher than that from GO0 and GO30.

After three-year river experience, the splitting tensile strengths for GO0, GO30, GO60 and GO90 were reduced to 3.88 MPa, 4.13 MPa, 5.22 MPa, and 4.77 MPa, respectively. The result, again, confirmed a degradation of mechanical strength after three-year river experience, and the reduction was less when higher amount of GO was incorporated. To be detailed, after river experience, the tensile splitting strength of GO0, GO30, GO60 and GO90 was reduced by 12.22%, 13.59%, 8.26% and 7.02%, respectively. In line with the compressive strength and the flexural strength, 0.06 wt.% GO could still be regarded as the optimum incorporation concentration based on splitting tensile strength, as the mix proportion could not only help attain the highest splitting tensile strength after standard curing, but also could maintain the splitting tensile strength to the highest extent after three-year river experience.

Normal distribution and Student’s *t* tests were performed again to examine the significances of difference (see Table 6), and the revealed trends complied with that based on compressive strength and that based on flexural strength.

In summary, with the incorporation of GO, the mortar specimens prepared in this study presented promising durability and mechanical properties. Combining early-age water permeation tests, chloride penetration tests and MIP tests, GO incorporation was validated to be effective in refining the microstructure and transport properties of cementitious composites. Also, combining dual CT scan and mechanical tests, GO incorporation was proven useful in retarding the degradation on durability and mechanical properties for cementitious composites serving underwater, so the application of GO acquired the potential to prolong the service life of marine infrastructure.

It should be highlighted that the underwater experiment in this study was carried out in river from real circumstance. Unlike simulated solution in laboratory, other factors, such as shear force caused by water flowing, seasonal temperature fluctuating, could all be considered in the present study, so the obtained results tended to be more compatible with that for the in-site marine infrastructure. Two differences can be drawn from in-site and laboratory tests. First, in laboratory, steady leaching solution was commonly used. As water flow was omitted, the effect of water action on concrete was overlooked. However, for in-site applications, since structures were subjected to a flowing underwater environment, the effect of water action, worked together with leaching to threat the durability of the structures. Second, laboratory tests observed high leaching degree near the sample surface, while for in-site occasions, even after three-year river experience, the leaching near the sample surface was incomplete (see Figure 10). The difference was resulted from the solution specimens were exposed to during tests. In the laboratory, accelerated leaching solutions were commonly used, and the leaching degree was thus high; while for in-site tests, since structures were exposed to water, the leaching progress was relatively slower. As a conclusion, laboratory results exaggerated the effect of leaching, while underestimated the effect of water actions on concrete durability. In this study, both effects were considered, and the revealed degradation mechanism was thus applicable for in-site concretes serving under different rivers.

According to our study, the optimum concentration for GO incorporation was selected to 0.06 wt.%, under which concentration the microstructure and transport properties for cementitious composites could be effectively refined, and to the largest extent the durability and mechanical properties could be retained after years of serving underwater.

## 4. Conclusions and Future Work

### 4.1. Conclusions

In this study, systematic work was carried out to understand the effect of graphene oxide (GO) incorporation on the durability and mechanical properties of cementitious composites serving underwater. Since the incorporated GO contents were small (less than 0.09 wt.%), and cement was partly blended by fly ash (byproduct of coal industry) in this study, the material costs were lower as compared to that using conventional construction materials. Combining water permeation test, chloride migration test and mercury intrusion porosimetry (MIP) test, the effects of GO incorporation on transport behaviors and microstructure were studied. Based on a novel dual-scan method, the spatial distribution of local porosity for mortars after three-year river experience was investigated. Furthermore, through mechanical tests, the initial and remaining strength for mortars before and after river experience were compared. The work highlights the importance to carry out degradation tests of concrete under in-site underwater environment, which will be of high reference value for the designing of marine infrastructure with promising durability and mechanical properties. Also, current work can help promote the application of nanomaterials in civil engineering. Several conclusions can be drawn from the present study.

(1) With the addition of GO nanoparticles, due to blocking of the permeation paths and increase on the crack tortuosity, the water permeation coefficient of mortar was reduced slightly during early curing age.
(2) The chloride migration coefficients for standard-cured mortars were reduced by the range of 4.98–9.54% with the addition of GO, based on which GO incorporation was proven effective in confining the transport behaviors of mortar.
(3) Based on MIP, the average open porosity of mortar, which controlled the ion migration properties of cementitious composites, would be reduced by the range of 3.37–11% with the incorporation of GO. Besides, the most probable pore radius would also be reduced after GO addition. Both results confirmed that GO addition refined the pore structure of mortar.
(4) Based on dual CT scan, the local porosity for non-leached mortar was approximately 15%, while the local porosity for mortars after river experience can increased to as high as 30%. Compared with MIP, the porosity obtained through dual CT scan was closer to the total porosity of mortar, so the porosities here determined both the mechanical and ion migration properties of the tested specimens. The incorporation of GO cannot only improve the leaching resistance, but also enhance the cracking resistance of mortar, both of which can improve the durability and mechanical properties of mortars serving underwater.
(5) With the addition of GO, the compressive strengths of standard-cured specimens could be improved by the range of 4.37–9.82%. Also, after a three-year river experience, the higher remaining compressive strengths were recorded for GO-incorporated mortars.
(6) The flexural strengths of standard-cured mortars could be improved by the range of 7.78–22.33% with the addition of GO. Besides, the remaining flexural strengths of mortars were less reduced with the addition of GO.
(7) The splitting tensile strengths of standard-cured mortars could be enhanced by the range of 8.14–28.73% when GO was added. Meanwhile, the splitting tensile strengths could on a larger extent be retained after three-year river experience for GO-incorporated mortars.
(8) Judging from transport properties, microstructure and mechanical properties for mortars before and after three-year river experience, the optimum mix proportion for GO was determined to be 0.06 wt.% in the study.

## 4.2. Future Work

The work in this study was compatible to concrete structures serving permanently underwater, such as deep-sea concrete structures, or underground concrete aqueducts. Since the studied structures were maintained at a saturated state throughout the test, the effect of wet-dry cycling on concrete degradation was insignificant. However, for many marine environments, especially those near the splash zones, wet-dry cycling can cause seasonal fluctuation of humidity inside concrete, which will significantly threat the durability performance. Therefore, to be systematic, future work is required to understand the coupled effects of leaching, water flushing as well as wet-dry cycling on the degradation mechanism of concrete. This work was based on mortar, while in real circumstances, concretes are more commonly used. To further understand the degradation mechanism of construction in-site, concrete should be adopted for study in future. All above mentioned work continues in our laboratory.

## Figures and Tables

**Table 1:** Chemical composition of cement and fly ash/wt%.

Binder	CaO	SiO_2_	Al_2_O_3_	Fe_2_O_3_	MgO	SO_3_	Others	LOI	Total
Cement	62.60	21.35	4.67	3.31	3.08	2.25	1.29	1.45	100.00
Fly ash	8.38	47.96	30.46	5.91	2.60	1.32	3.37	-	100.00

**Table 2:** Chemical and physical properties of graphene oxide (GO).

Purity	Carbon Content (%)	Oxygen Content (%)	Flake Φ (μm)	Thickness (nm)
97%	46	53	0.2–10	1

**Table 3:** Mix proportions for GO-incorporated mortars ($\mathrm{kg}/m^{3}$).

Group	Cement	Fly Ash	Quartz Sand	Water	GO
**GO0**	613	263	438	263	0
**GO30**	613	263	438	263	0.2628
**GO60**	613	263	438	263	0.5256
**GO90**	613	263	438	263	0.7884

**Table 4:** Significances of difference based on Student’s *t* test (examining compressive strength).

Groups Compared	Significance before River Immersing	Significance after River Immersing
**GO0-GO30**	0.179	0.313
**GO0-GO60**	0.001	0.002
**GO0-GO90**	0.064	0.015

**Table 5:** Significances of difference based on Student’s *t* test (examining flexural strength).

Groups Compared	Significance before River Immersing	Significance after River Immersing
**GO0-GO30**	0.036	0.647
**GO0-GO60**	0.000	0.003
**GO0-GO90**	0.003	0.010

**Table 6:** Significances of difference based on Student’s *t* test (examining splitting tensile strength).

Groups Compared	Significance before River Immersing	Significance after River Immersing
**GO0-GO30**	0.147	0.345
**GO0-GO60**	0.000	0.003
**GO0-GO90**	0.013	0.008
